# *QuickStats:* Prevalence* of Anemia^†^ Among Adults Aged ≥65 Years, by Sex and Age Group — National Health and Nutrition Examination Survey, 2013–2016

**DOI:** 10.15585/mmwr.mm6742a8

**Published:** 2018-10-26

**Figure:**
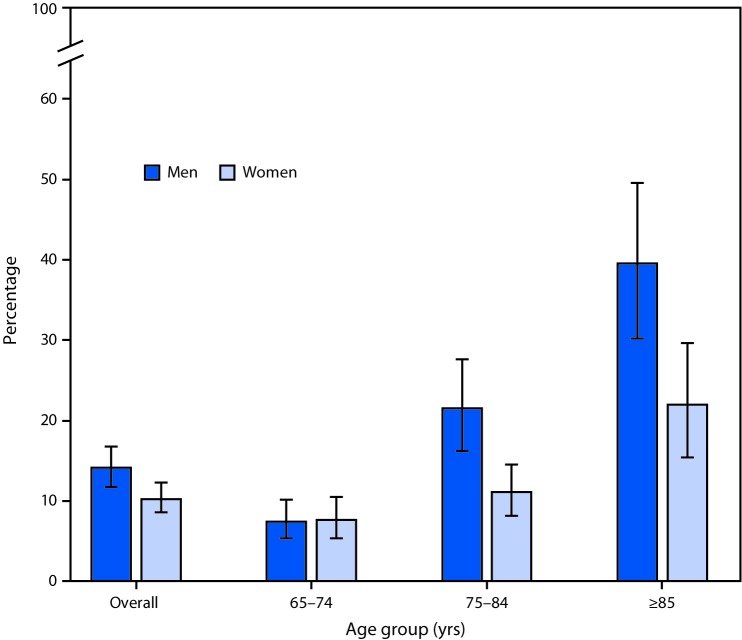
During 2013–2016, the prevalence of anemia among persons aged ≥65 years increased with increasing age for both men and women. Among men, the prevalence increased from 7.4% for those aged 65–74 years to 39.5% for those aged ≥85 years. The percentage of women with anemia increased from 7.6% for those aged 65–74 years to 21.9% for those aged ≥85 years. The prevalence of anemia was higher for men compared to women among those aged 75–84 years and those aged ≥85 years.

**Sources:** National Health and Nutrition Examination Survey, 2013–2016. https://www.cdc.gov/nchs/nhanes/index.htm; Seitz AE, et al. Anemia prevalence and trends in adults aged 65 and older: U.S. National Health and Nutrition Examination Survey: 2001–2004 to 2013–2016. https://onlinelibrary.wiley.com/doi/epdf/10.1111/jgs.15530.

